# Supplementary material for: Integrated Transcriptomic and Metabolomics Analyses Reveal Molecular Responses to Cold Stress in Coconut (Cocos nucifera L.) Seedlings
Source: Int J Mol Sci. 2023 Sep 26;24(19):14563. doi: 10.3390/ijms241914563 (PMC10572742; doi:10.3390/ijms241914563)
Supplement: Supplementary file 1 [file ijms-24-14563-s001.zip › Supplementary Material.pdf]

Supplementary Material

Integrated transcriptomic and metabolomics analyses reveal molecular responses to cold stress in coconut (*Cocos nucifera* L.) seedlings

Lilan Lu [1,†], Weibo Yang [1,†], Zhiguo Dong [1], Longxiang Tang [1], Yingying Liu [2], Shuyun Xie [2,*] and Yaodong Yang [1,*]

1 Hainan Key Laboratory of Tropical Oil Crops Biology/Coconut Research Institute, Chinese Academy of Tropical Agricultural Sciences, Wenchang 571339, China; lulilan1234@163.com (L.L.); yangweibo623@126.com (W.Y.); dongzg@catas.cn (Z.D.); tanglx@catas.cn (L.T.)

2 School of Earth Sciences, China University of Geosciences, Wuhan 430074, China; yingying1795@126.com

* Correspondence: tinaxie@cug.edu.cn (S.X.); yyang@catas.cn (Y.Y.)

† These authors contributed equally to this work.

1 Supplementary Data

1.1 Supplementary Figures

Figure S1. **Gene expression analysis in LT_{30} vs. CK_{30}.** (a) The gene expression distribution in LT_{30} vs. CK_{30}. (b) Correlation analysis of the genes in LT_{30} and CK_{30}. (c) Principal component analysis (PCA) of expressed genes in LT_{30} and CK_{30}. (d) Differentially expressed genes (DEGs) volcano map in LT_{30} vs. CK_{30}. Each point represents a gene, and the X-axis represents the logarithm of the expression difference multiple of a certain gene in the two samples. The Y-axis represents the negative value of the statistical significance of the change in gene expression. The green dots represent up-regulated DEGs, the red dots represent down-regulated DEGs, and the black dots represent non-differentially expressed genes. (e) Statistics of up-regulated and down-regulated DEGs.

Figure S2. **Classification of GO annotation of differentially expressed genes (DEGs) in LT_{30} vs. CK_{30}.** (a) GO annotation classification of up-regulated DEGs. (b) GO annotation classification of down-regulated DEGs.The X-axis is the GO classification, the left side of Y-axis is the percentage of the gene number, and the right side of the Y-axis is the gene number.

Figure S3. **GO enrichment site map of differentially expressed genes (DEGs) in LT_{30} vs. CK_{30}.** The X-axis is generatio, that is the proportion of the genes of interest in the entry to all the differentially expressed genes, and the Y-axis is each GO annotation entry. The size of the dots represents the number of DEGs annotated in the pathway, and the color of the dots represents the q value of the hypergeometric test. (a) Biological process of up-regulated DEGs. (b) Cellular component of up-regulated DEGs. (c) Molecular function of up-regulated DEGs. (d) Biological process of down-regulated DEGs. (e) Cellular component of down-regulated DEGs. (f) Molecular function of down-regulated DEGs.

Figure S4. **KEGG classification map of differentially expressed genes (DEGS) in LT_{30} vs.**

CK_{30}.The Y-axis is the name of the KEGG metabolic pathway, and the X-axis is the number of genes annotated to the pathway and their proportion to the total number of annotated genes.(a) All DEGs.(b) Up-regulated DEGs.(c) Down-regulated DEGs.

Figure S5. **KEGG pathway rich distribution map of differentially expressed genes (DEGs) in LT_{30} vs. CK_{30}**. Each circle represents a KEGG pathway, the Y-axis represents the pathway name, and the X-axis represents enrichment factor, which represents the ratio of the proportion of differential genes annotated to a pathway to the proportion of all genes annotated to that pathway. The color of the circle represents qvalue, which is the P value after the multiple hypothesis testing correction. The size of the circle indicates the number of genes enriched in the pathway.(a) All DEGs.(b) Up-regulated DEGs.(c) Down-regulated DEGs.

Figure S6. Expression patterns of 19 DEGs of coconut seedling assessed by qRT-PCR in LT_{30} vs. CK_{30}.(a) Expression patterns of 19 DEGs in LT_{30} vs. CK_{30}. (b) Comparison of the log_2Fold change (LT_{30}/CK_{30}) from 19 selected genes by RNA-seq and qRT-PCR in 3 biological replicates.

Figure S7. **Differential accumulated metabolite (DAMs) analysis in LT_{30} vs.CK_{30}**. (a, d) Volcanic map of DAMs in the positive and negative ion modes. Each point represents a metabolite, the X-axis represents the multiple change of each substance in the group (taking the logarithm base 2), the Y-axis represents the P-value of the T-test (taking the logarithm base 10), and the scatter point size represents the VIP value of the OPLS-DA model. Blue dots represent up-regulated DAMs, red dots represent down-regulated DAMs, and gray represents metabolites that are detected but not significantly different. In addition, the first 5 metabolites qualitatively identified were selected and labeled in the figure after ranking by P-value.（b, e）OPLS-DA score chart in the positive and negative ion modes.The X-axis (t1) represents the prediction component (the inter-group variance component), the Y-axis (t2) represents the orthogonal component (the intra-group variance component), and the transverse Y-axis percentage represents the component's share in the total variance. Below the figure are the parameters of the model, including R_2X, R_2Y, Q_2Y, RMSEE (root mean square error), pre (number of predicted components), ort (number of orthogonal components).(c, f) Difference multiples bar chart of top10 up-regulated and down-regulated DAMs in the positive and negative ion modes. The label of each column indicates the metabolite name, which is distinguished by the top down, the upward upward is red, the downward is green, and the column length represents log_2FC.

Figure S8. **KEGG database classification of metabolites in LT_{30} vs. CK_{30}**.The entries under the same box in the figure represent the level classification notes of the KEGG pathway, corresponding to KO pathway Level 1 and KO Pathway Level 2. The length of the column represents the amount of metabolites annotated by the pathway. (a) In the positive ion modes. (b) In the negative ion modes.

Figure S9. **Comprehensive analysis of metabolomics and transcriptomics of coconut seedlings under cold stress in LT_{30} vs. CK_{30}** .(a, d) Hierarchical clustering heat map of differentially expressed genes (DEGs) and differential accumulated metabolites (DAMs) correlation analysis in the positive and negative ion modes. Each column in the hierarchical clustering heat map represents a sample of the difference group, and each row represents a significant difference gene or significant difference metabolite.(b, e) Nine quadrant diagrams

in the positive and negative ion modes. (c, f) KEGG enriched bubble diagram of top 30 DEGs/DAMs in the positive and negative ion modes.The X-axis of enrich factor represents the enrichment factor (Diff/Background) of the pathway in different omics, and the Y-axis represents the name of the KEGG pathway. The gradient of red to blue represents the change of the significance degree of enrichment from high to low, which is represented by Pvalue. The shapes of the bubbles represent different omics, with circles representing the transcriptome and triangles representing the metabolome; The size of the bubble represents the number of different metabolites or genes.

Figure S10. (a, b) Correlation network diagram of differentially expressed genes (DEGs) and differential accumulated metabolites (DAMs) in LT_{30} vs. CK_{30}. The circles represent metabolites, the boxes represent genes, the numbers on the line represent correlation coefficients, positive correlations are red, negative correlations are green, and the larger the correlation coefficients, the wider the lines and the darker the colors.

Figure S11.Comprehensive analysis of metabolomics and transcriptomics of coconut seedlings under cold stress in biosynthesis of other secondary metabolites and metabolism of cofactors and vitamins. Including the pathways such as (a) changes of DEGs in flavonoid biosynthesis, (b) changes of DEGs in isoquinoline alkaloid biosynthesis, (c) changes of DEGs in stilbenoid, diarylheptanoid and gingerol biosynthesis, (d) changes of DEGs in folate biosynthesis, (e) changes of DEGs in flavone and flavonol biosynthesis, (f) changes of DEGs in tropane, piperidine and pyridine alkaloid biosynthesis, (g) Changes of DEGs in isoflavonoid biosynthesis, (h) Changes of DEGs in ubiquinone and other terpenoid-quinone biosynthesis, (i) changes of DEGs in nicotinate and nicotinamide metabolism, (j) Changes of DEGs in vitamin B6 metabolism.(k) changes of DEGs in phenylpropanoid biosynthesis. (l) changes of DEGs in biotin metabolism. (m) Changes in metabolites associated with these pathways.

Figure S12.Comprehensive analysis of metabolomics and transcriptomics of coconut seedlings under cold stress in translation and membrane transport. Including the pathways such as (a) changes of DEGs in aminoacyl-tRNA biosynthesis, (b) changes of DEGs in ABC transporters. (c) Changes in metabolites associated with these pathways.

1.2 Supplementary Tables

Table S1. The dry weight and plant height under under LT and CK treatments.

Table S2. Summary of the sequencing data generated for RNA-seq and mapping of the apple genome under LT_{30} and CK_{30} treatments.

Table S3. (a) 23795 genes with FPKM value in RNA-seq under LT_{30} and CK_{30}. (b) 11591 DEGs in RNA-seq under LT_{30} and CK_{30}.(c) 9968 DEGs annotation in RNA-seq in LT_{30} vs.CK_{30}.

Table S4. Analyses of top 20 Gene ontology (Go) enrichment pathways in LT_{30} vs.CK_{30}.

Table S 5. (a) KEGG enrichment pathways based on 884 DEGs in LT_{30} vs.CK_{30}.(b) Candidate genes related to main KEGG enrichment pathways based on 9968 DEGs in LT_{30} vs.CK_{30}.

Table S6. Summary of the core genes and transcription factors in LT_{30} vs.CK_{30}.

Table S7. 648 Metabolites identified in coconut leaves in LT_{30} vs.CK_{30} (pos.(423)+neg.(225))

Table S8. (a) 206 different accumulated metabolites(DAMs) in positive ion mode identified in coconut leaves in LT_{30} vs.CK_{30}. (b) 97 different accumulated metabolites (DAMs) in negative ion mode identified in coconut leaves in LT_{30} vs.CK_{30}.

Table S9. (a) KEGG enrichment pathways of 52 different accumulated metabolites(DAMs)from 92 mtabolites in positive ion mode in LT_{30} vs.CK_{30}. (b) KEGG enrichment pathways of 34 different accumulated metabolites(DAMs)from 72 mtabolites in negative ion mode in LT_{30} vs.CK_{30}. (c) Different accumulated metabolites(DAMs) of KEGG enrichment pathways in positive ion mode in LT_{30} vs.CK_{30}. (d) Different accumulated metabolites(DAMs) of KEGG enrichment pathways in negative ion mode in LT_{30} vs.CK_{30}.

Table S10. (a) 47 KEGG enrichment pathways of correlations between differentially expressed genes (DEGs) and differentially accumulated metabolites (DAMs) in positive ion mode in LT_{30} vs.CK_{30}. (b) 31 KEGG enrichment pathways of correlations between DEGs and DAMs in negative ion mode in LT_{30} vs.CK_{30}.

Table S11. (a) DEGs of KEGG enrichment pathways of correlations between differentially expressed genes (DEGs) and differentially accumulated metabolites (DAMs) in LT_{30} vs.CK_{30}.(in positive and negative ion modes).(b) DAMs of KEGG enrichment pathways of correlations between differentially expressed genes (DEGs) and differentially accumulated metabolites (DAMs) in LT_{30} vs.CK_{30} (in positive and negative ion modes).

Table S12. Primers used in qRT-PCR validation under LT_{30} andCK_{30} treatments.
