# Supplementary material for: Integrated Transcriptomic and Metabolomics Analyses Reveal Molecular Responses to Cold Stress in Coconut (Cocos nucifera L.) Seedlings
Source: Int J Mol Sci. 2023 Sep 26;24(19):14563. doi: 10.3390/ijms241914563 (PMC10572742; doi:10.3390/ijms241914563)
Supplement: Supplementary file 1 [file ijms-24-14563-s001.zip › Table S1.pdf]

Table S1. The dry weight and plant height LT and CK treatments.

**Treatment**	**Treatment Time (Days)**	**Plant height（cm）**	**Dry weight(g/plant) M±SD**
CK	0	60.70±2.36 a	65.67±0.52 a
	10	75.70±2.83 a	76.33±0.61 a
	20	90.70±2.66 b	88.67±0.74 b
	30	105.78±2.36 c	112.65±0.45c
LT	0	60.70±2.36 a	65.67±0.52 a
	10	65.70±2.45 a	68.39±0.26 a
	20	70.70±2.65 a	70.67±0.36 a
	30	80.70±2.16 a	75.57±0.66 a

Means in the same category followed by different letters indicate significant differences at $p< 0.05$ using student's t-test. The data represent the means of replications (n=3)±SD.
