# Supplementary figures and images for: Integrated Transcriptomic and Metabolomics Analyses Reveal Molecular Responses to Cold Stress in Coconut (Cocos nucifera L.) Seedlings
Source: Int J Mol Sci. 2023 Sep 26;24(19):14563. doi: 10.3390/ijms241914563 (PMC10572742; doi:10.3390/ijms241914563)

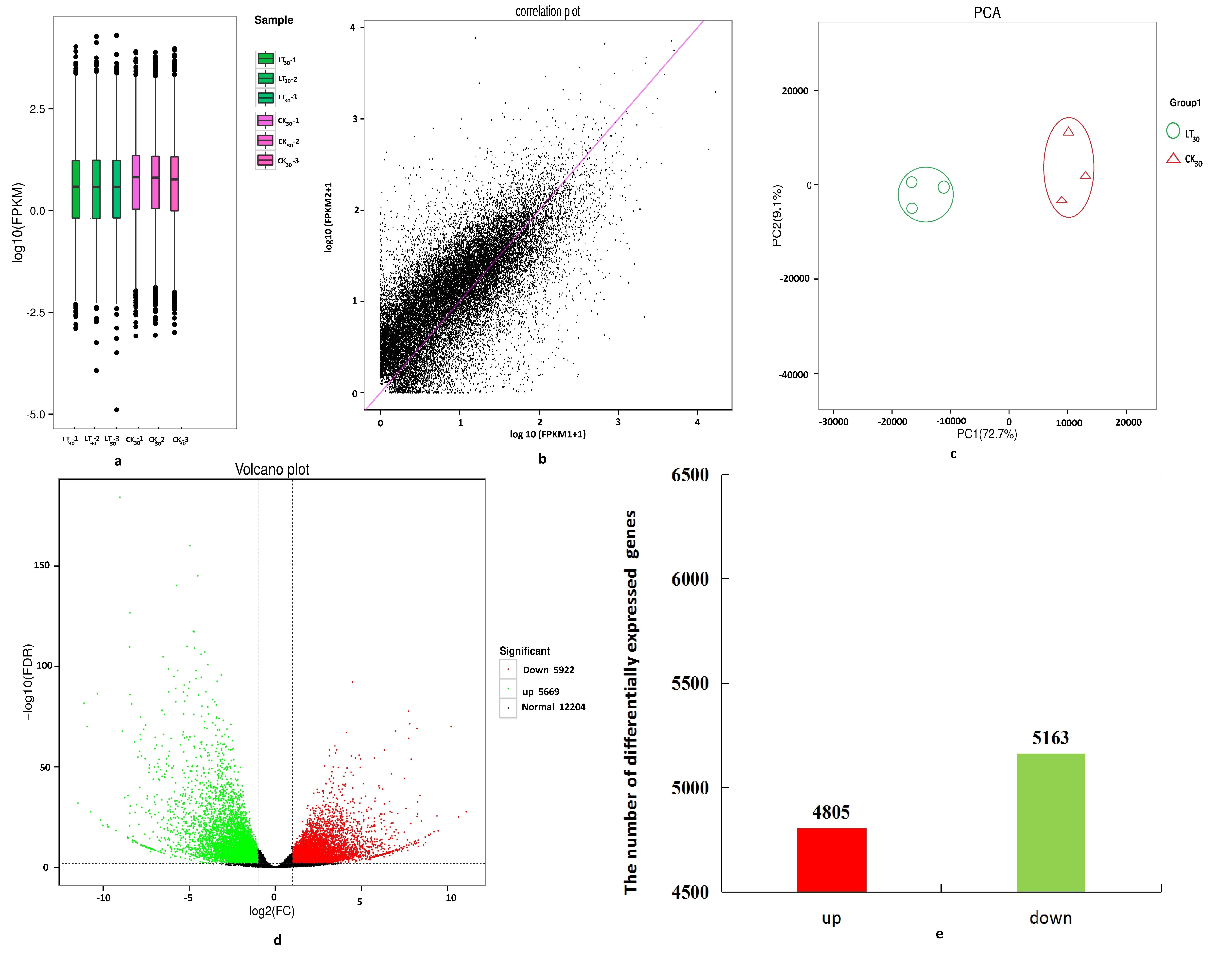

Supplement: Supplementary file 1 [file ijms-24-14563-s001.zip › ╕╜═╝/Figure S1.jpg]

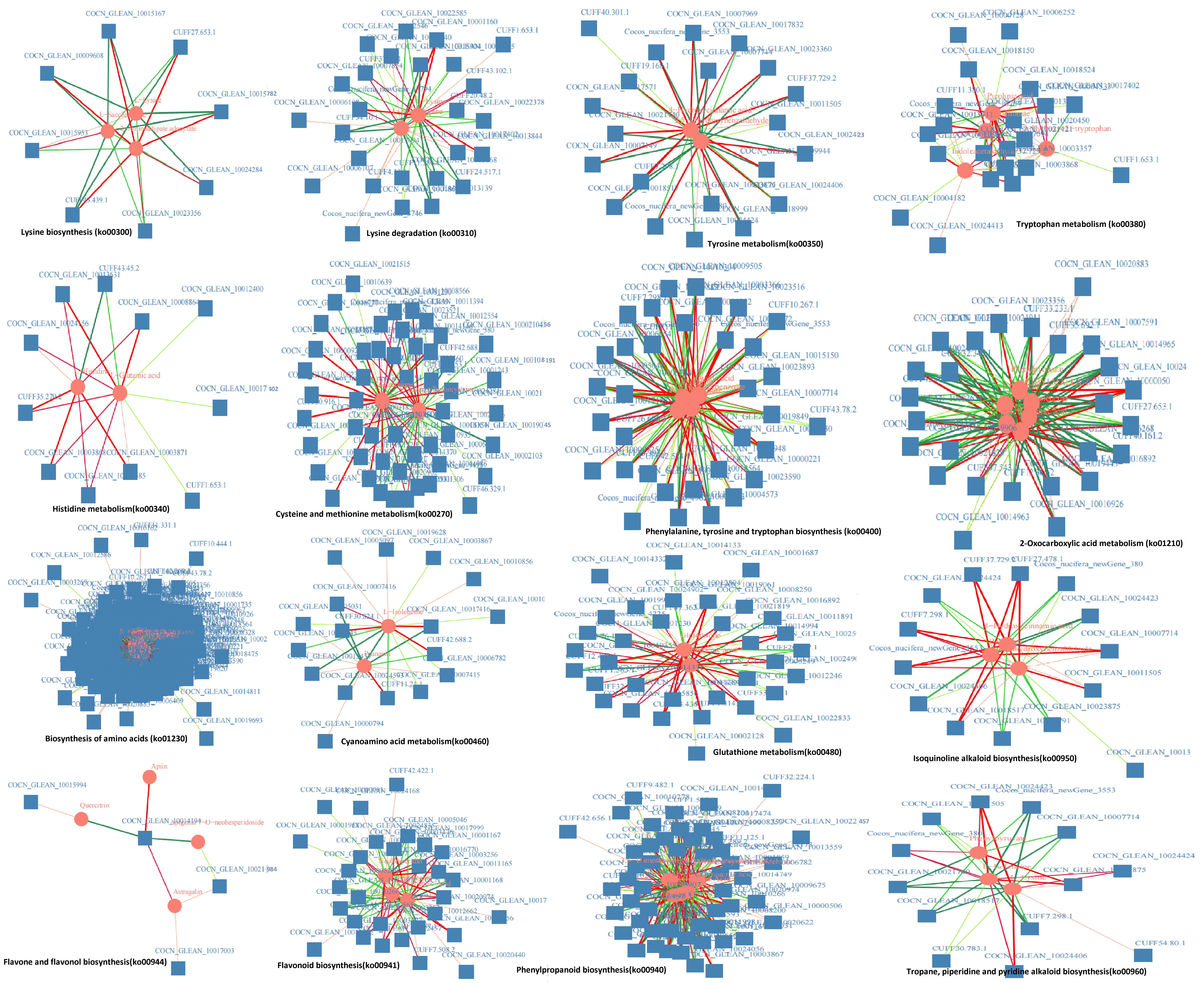

Supplement: Supplementary file 1 [file ijms-24-14563-s001.zip › ╕╜═╝/Figure S10a.jpg]

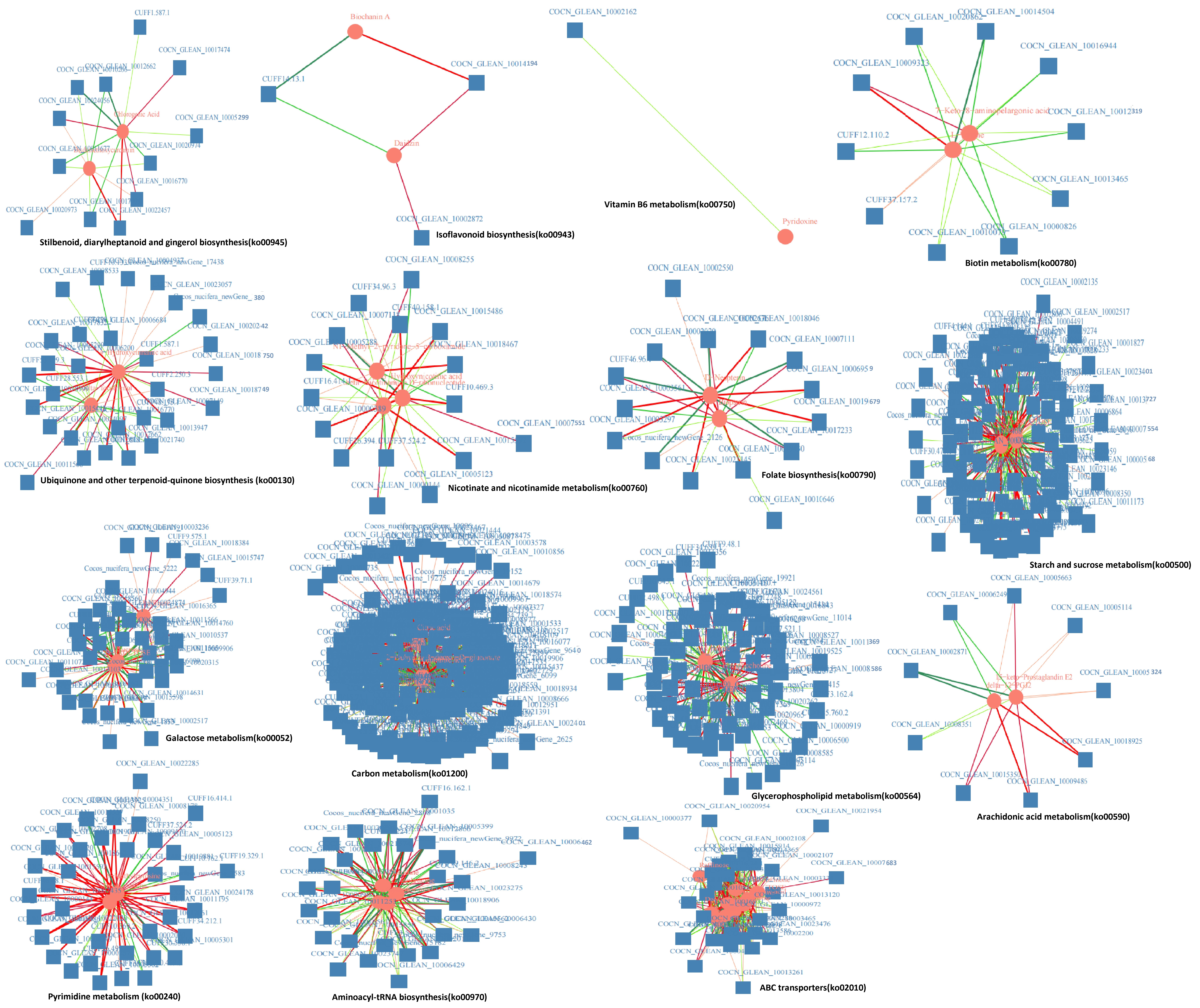

Supplement: Supplementary file 1 [file ijms-24-14563-s001.zip › ╕╜═╝/Figure S10b.jpg]

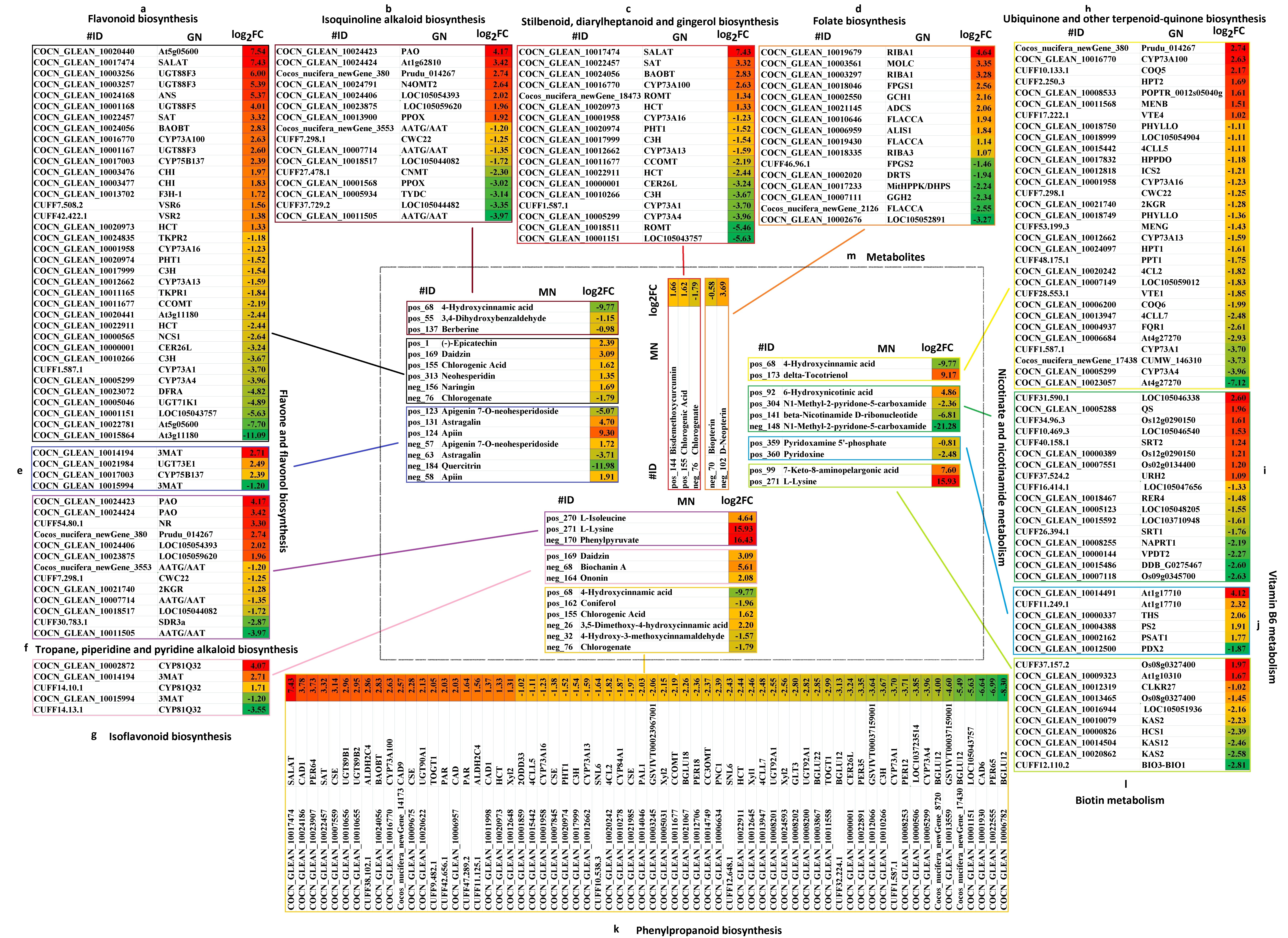

Supplement: Supplementary file 1 [file ijms-24-14563-s001.zip › ╕╜═╝/Figure S11.jpg]

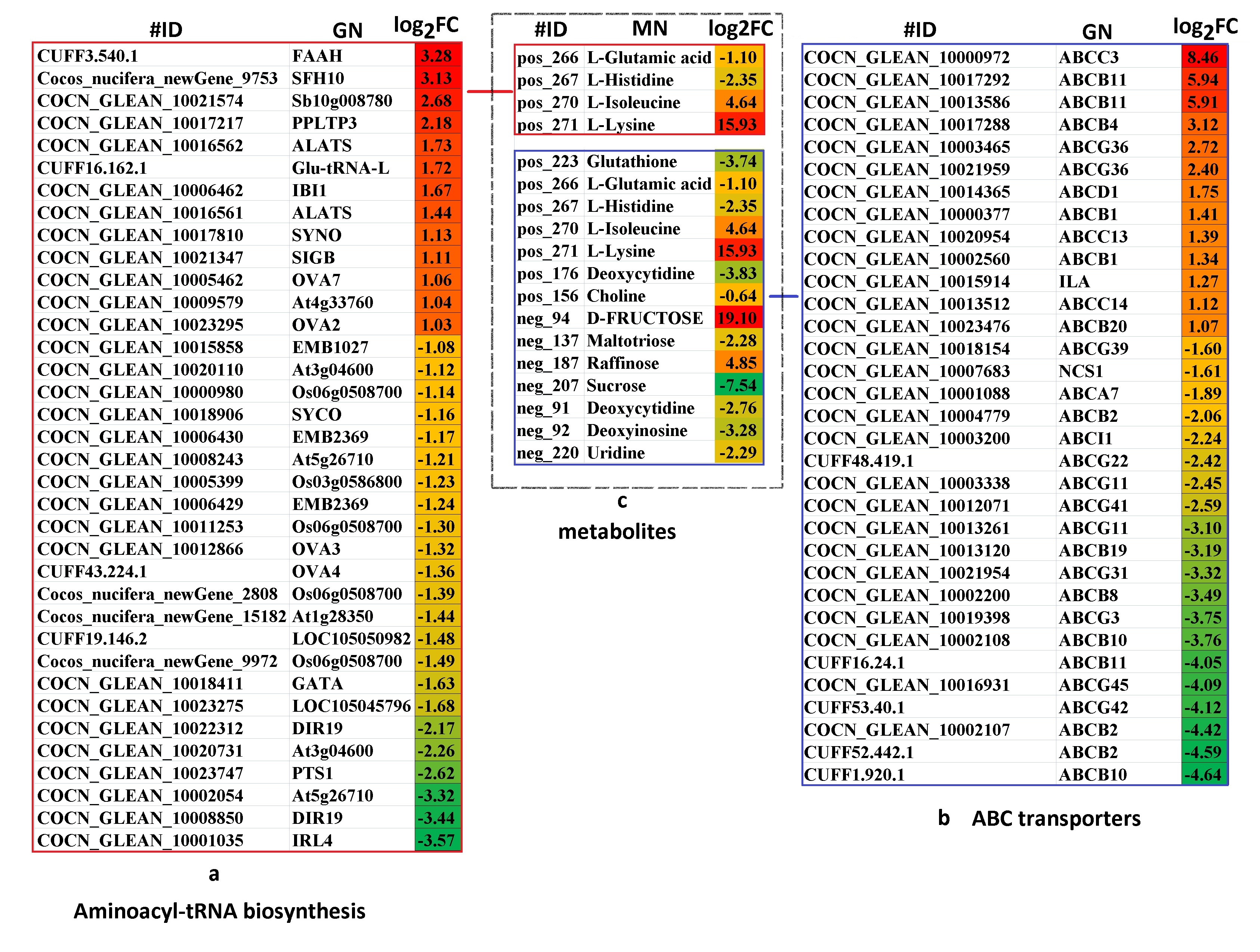

Supplement: Supplementary file 1 [file ijms-24-14563-s001.zip › ╕╜═╝/Figure S12.jpg]

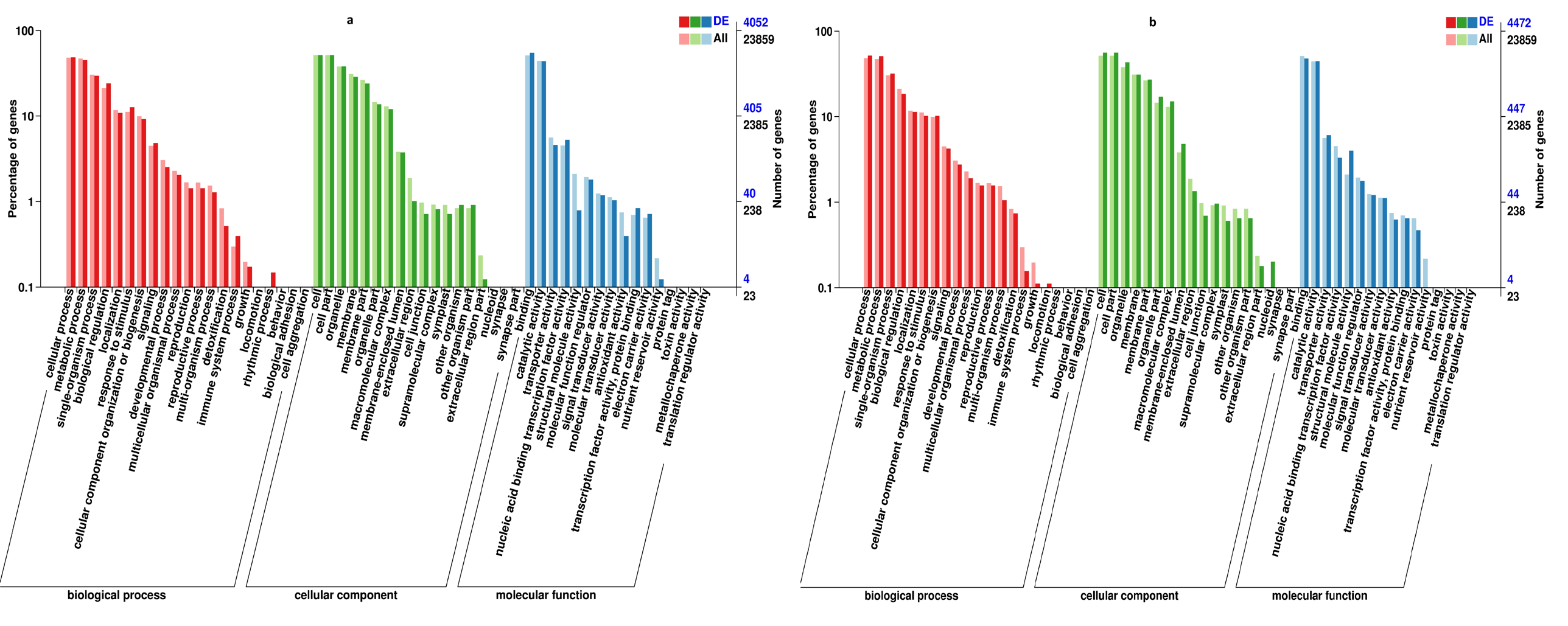

Supplement: Supplementary file 1 [file ijms-24-14563-s001.zip › ╕╜═╝/Figure S2.jpg]

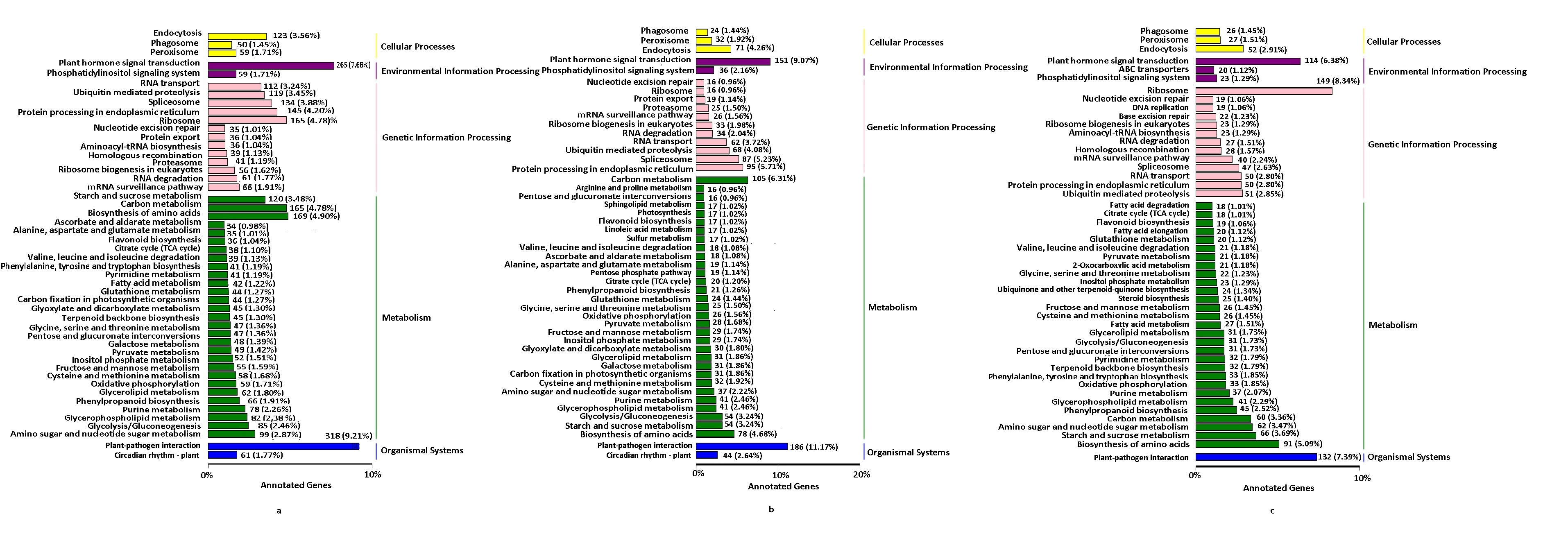

Supplement: Supplementary file 1 [file ijms-24-14563-s001.zip › ╕╜═╝/Figure S4.jpg]

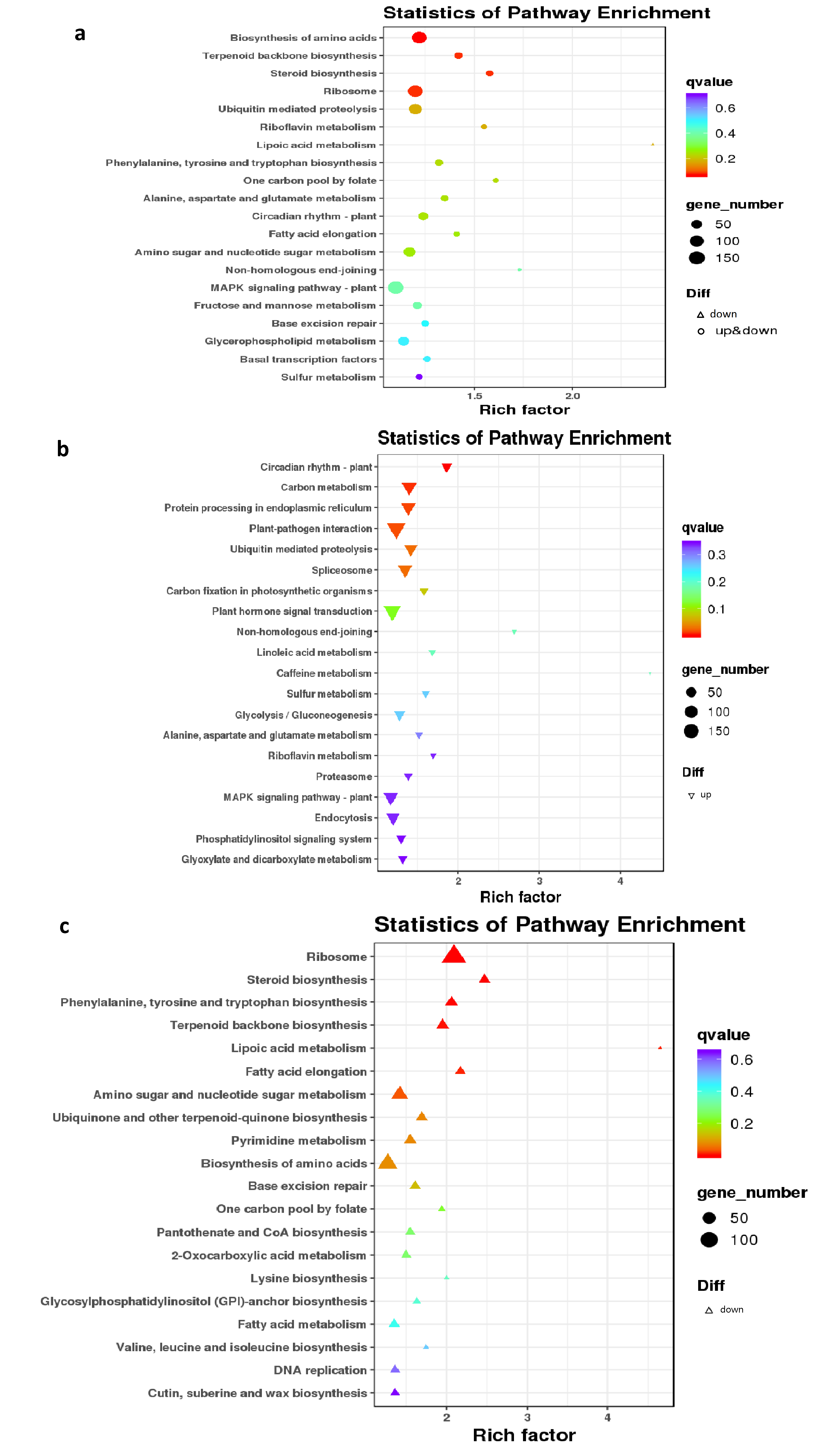

Supplement: Supplementary file 1 [file ijms-24-14563-s001.zip › ╕╜═╝/Figure S5.png]

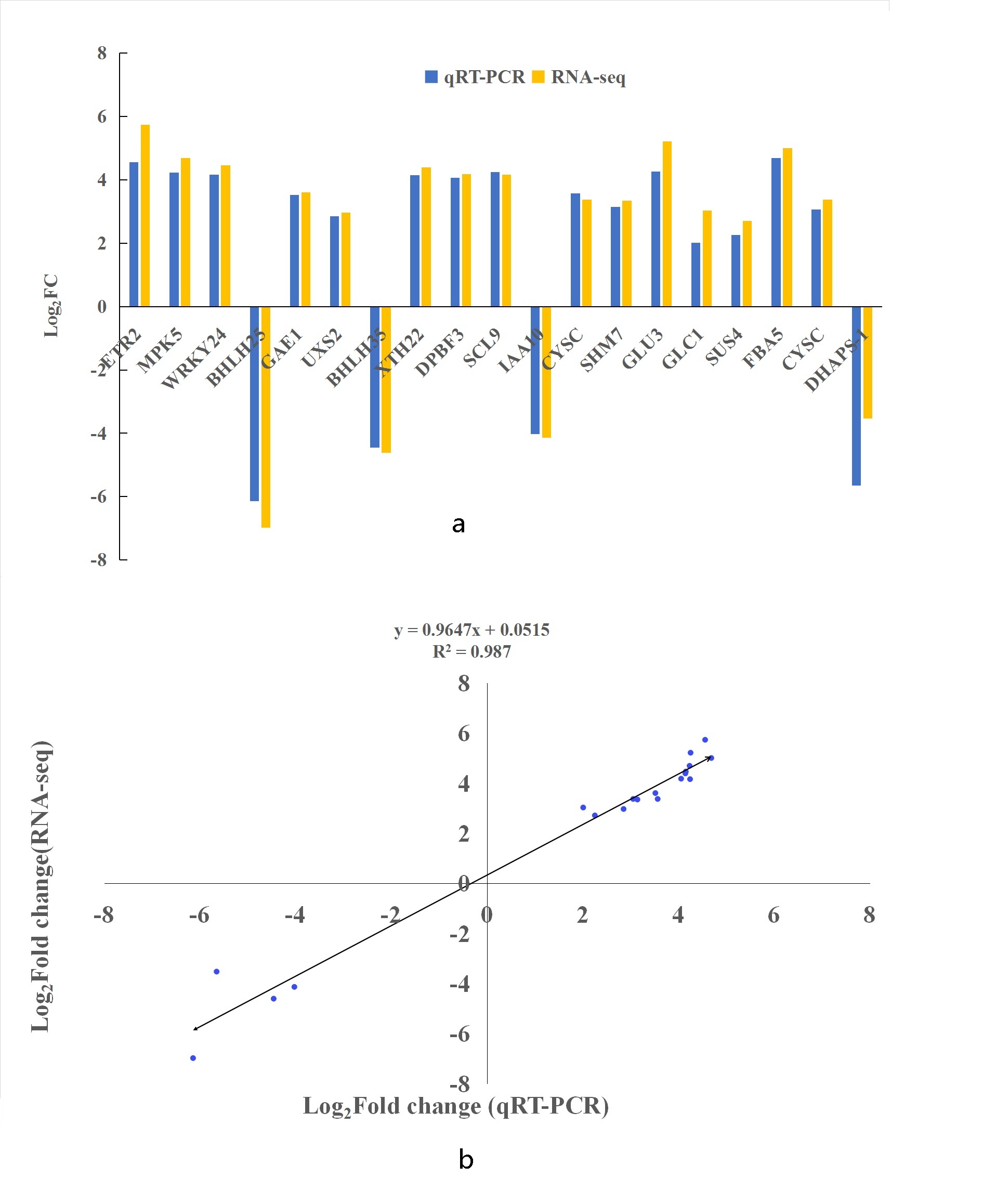

Supplement: Supplementary file 1 [file ijms-24-14563-s001.zip › ╕╜═╝/Figure S6.jpg]

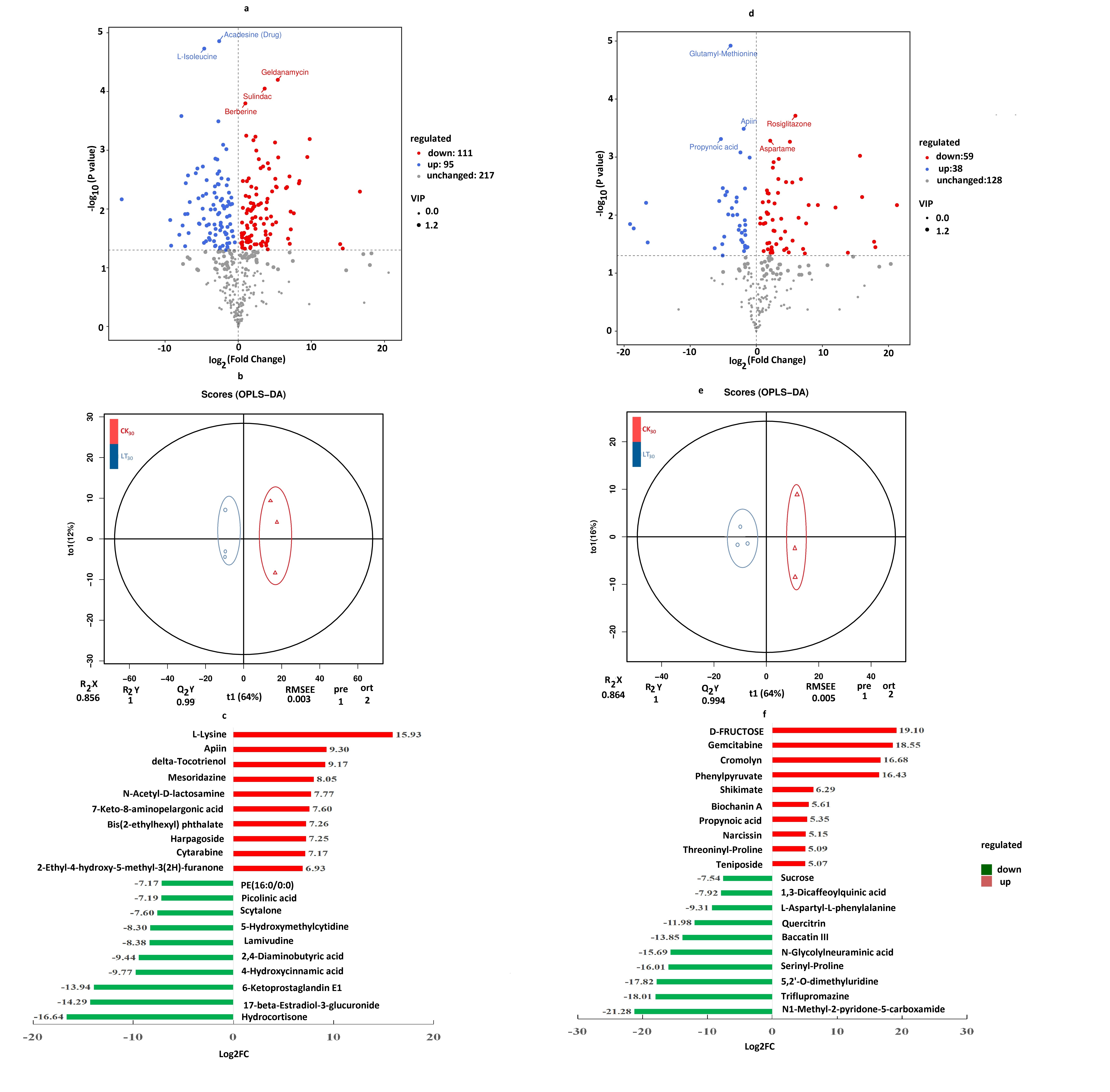

Supplement: Supplementary file 1 [file ijms-24-14563-s001.zip › ╕╜═╝/Figure S7.jpg]

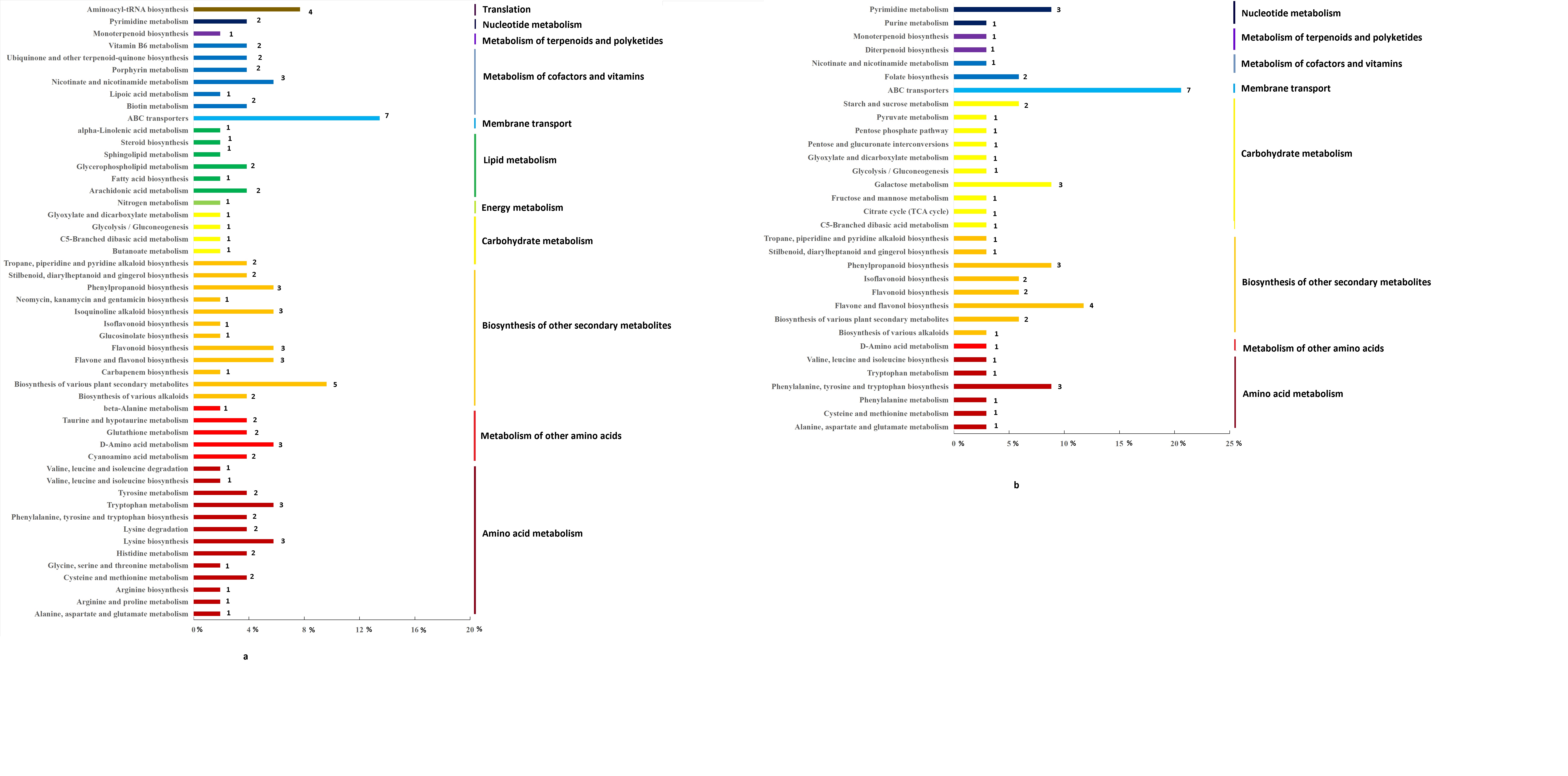

Supplement: Supplementary file 1 [file ijms-24-14563-s001.zip › ╕╜═╝/Figure S8.jpg]

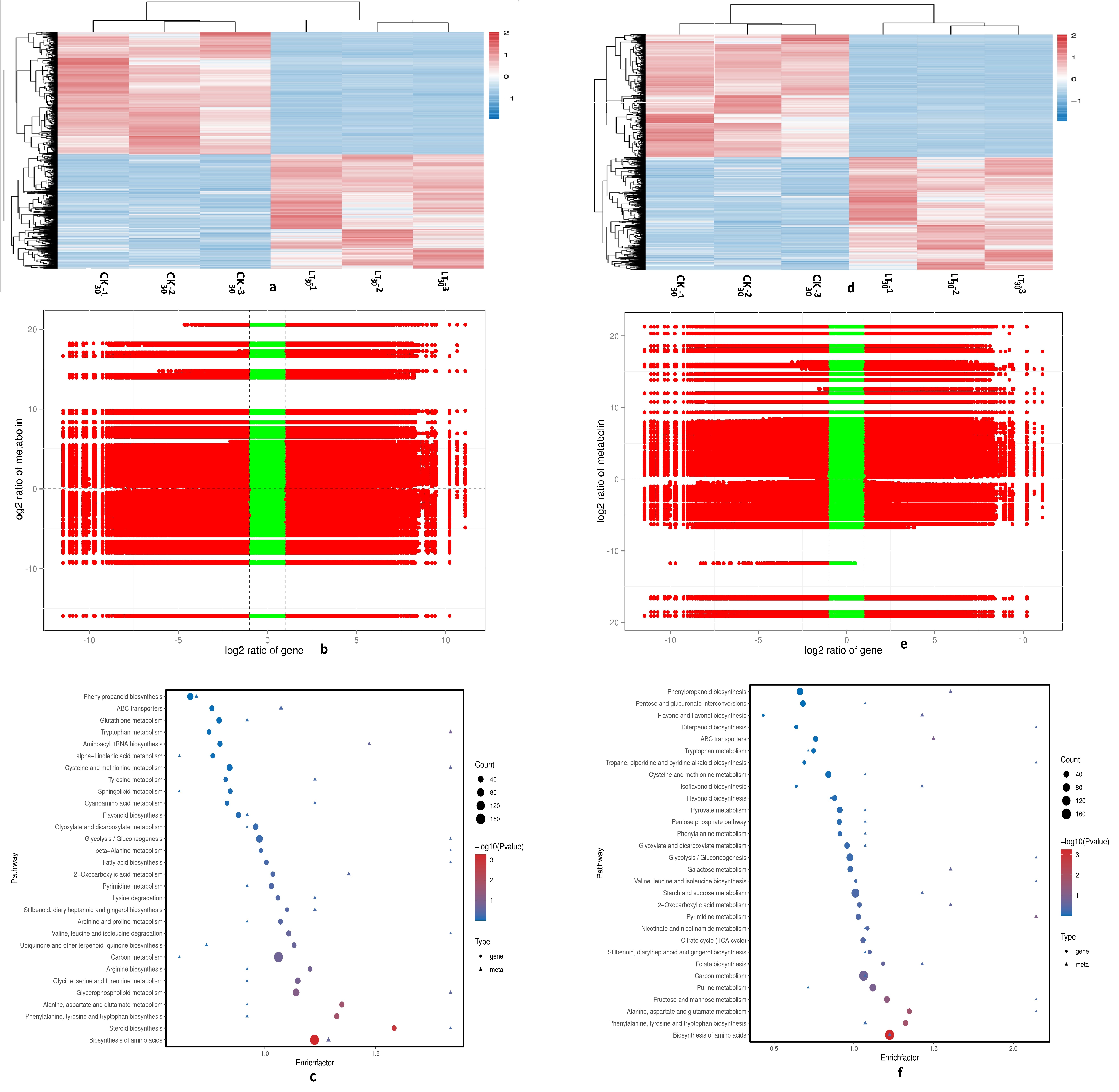

Supplement: Supplementary file 1 [file ijms-24-14563-s001.zip › ╕╜═╝/Figure S9.jpg]
